# Methods for Assessing Expiratory Flow Limitation during Tidal Breathing in COPD Patients

**DOI:** 10.1155/2012/234145

**Published:** 2012-09-02

**Authors:** Nickolaos G. Koulouris, Georgios Kaltsakas, Anastasios F. Palamidas, Sofia-Antiopi Gennimata

**Affiliations:** Respiratory Function Laboratory, 1st Department of Respiratory Medicine, Medical School, National and Kapodistrian University of Athens and “Sotiria” Chest Disease Hospital, 11527 Athens, Greece

## Abstract

Patients with severe COPD often exhale along the same flow-volume curve during quite breathing as during forced expiratory vital capacity manoeuvre, and this has been taken as indicating expiratory flow limitation at rest (EFL_T_). Therefore, EFL_T_, namely, attainment of maximal expiratory flow during tidal expiration, occurs when an increase in transpulmonary pressure causes no increase in expiratory flow. EFL_T_ leads to small airway injury and promotes dynamic pulmonary hyperinflation with concurrent dyspnoea and exercise limitation. In fact, EFL_T_ occurs commonly in COPD patients (mainly in GOLD III and IV stage) in whom the latter symptoms are common. The existing up-to-date physiological methods for assessing expiratory flow limitation (EFL_T_) are reviewed in the present work. Among the currently available techniques, the negative expiratory pressure (NEP) has been validated in a wide variety of settings and disorders. Consequently, it should be regarded as a simple, non invasive, most practical, and accurate new technique.

## 1. Introduction

Some experts use the term *chronic airflow limitation* as a synonym for chronic obstructive pulmonary disease (COPD) to indicate the reduction in maximum expiratory flow that occurs in this disease (and indeed in other pulmonary diseases). Patients with severe COPD often exhale along the same flow-volume curve during quite breathing as during forced expiratory vital capacity manoeuvre, and this has been taken as indicating flow limitation at rest (EFL_T_). Consequently, the term *tidal expiratory flow limitation* (EFL_T_) is used to indicate that maximal expiratory flow is achieved during tidal breathing at rest or during exercise. This is characteristic of intrathoracic flow obstruction. The former term does not imply that EFL_T_ actually occurs during tidal breathing [1]. The location of expiratory flow limitation is considered to be in the central airways (4th–7th generation) and move to the periphery during forced expiratory manoeuvres. It is located beyond the 7th (i.e., from the 8th onwards) generation during tidal breathing [2–4].

 Tidal expiratory flow limitation (EFL_T_) [5–8] plays a central role according to a recent hypothesis [5] on the transition from small airways disease (SAD) to overt COPD in smokers. EFL_T_ implies inhomogeneity of ventilation distribution with concurrent impairment of gas exchange and unevenly distributed stress and strain within the lung, which is amplified by tissue interdependence [6, 7] and may lead to *small airway* injury [5–8]. Initially, the latter is histologically characterized by denuded epithelium, rupture of alveolar-airway attachments, and increased number of polymorphonuclear leucocytes [5–7]. Studies in which heliox (80% He/20% O_2_) was administered in COPD patients provided also corroborative evidence that EFL_T_ was located in the peripheral airways [2–4]. EFL_T_ promotes dynamic pulmonary hyperinflation and PEEPi with concurrent dyspnoea and exercise limitation [8]. In fact, EFL_T_ occurs commonly in GOLD III and IV stage patients causing dynamic hyperinflation and severe dyspnoea [9]. It should be noted that the important role of expiratory flow limitation in COPD patients has been studied in a variety of clinical settings (during mechanical ventilation and exercise, correlation with dyspnoea, orthopnoea, and the other lung function indexes, before and after bronchodilatation, various postures).

## 2. Clinical Significance of EFL 

The important role of EFL_T_ in chronic dyspnoea and exercise impairment for a surprisingly wide range of clinical circumstances was enlightened by the techniques of detecting it, but mainly by the use of negative expiratory pressure (NEP) technique. EFL_T_ measured with the NEP technique is a much better predictor of chronic dyspnoea than FEV_1_, and FEV_1_ is not a specific predictor of EFL_T_ in COPD patients. These findings suggest that EFL_T_ measured by the NEP technique may be more useful in the evaluation of dyspnoea in COPD patients than spirometric measurements [10].

 The improvement of inspiratory capacity (IC) after bronchodilator administration [11], which is mainly limited to patients with EFL at rest and therefore usually exhibits a reduction of baseline IC, entails reduction in dyspnoea both at rest and during light exercise [12]. The fact that after bronchodilator administration there is a significant reduction of dynamic hyperinflation (DH) only in patients with EFL at rest further supports the usefulness of stratifying COPD patients in subgroups with and without EFL in order to predict an improvement in DH [11]. COPD patients with EFL may experience less breathlessness after a bronchodilator, at least during light exercise, than those without EFL. This beneficial effect, which is closely related to an increase in IC at rest, occurs even in the absence of a significant improvement of FEV_1_ [12]. Though, in the past, bronchodilator testing focused on changes of FEV_1_, the scrutiny of changes in IC in non-EFL_T_ and EFL_T_ COPD patients should provide useful information. In contrast, the detection of EFL_T_ did not predict the changes of EELV or dyspnoea occurring after bronchodilation [13].

Díaz et al. [14] found that IC was the only spirometric parameter, in which there was almost no overlap between non-EFL_T_ and EFL_T_ COPD patients. The non-EFL_T_ patients had almost all normal IC whilst the EFL_T_ all had <80% pred in a group of 52 COPD patients. Linear regression analysis performed separately for these EFL_T_ and non-EFL_T_ patients showed that in the EFL_T_ patients the sole predictor of exercise capacity was IC% pred, whilst in the non-EFL_T_ the ratio FEV_1_/FVC% pred was the sole predictor. Díaz et al. [15] also reported that in EFL_T_ COPD patients, the maximal tidal volume and hence maximal oxygen consumption are closely related to the reduced IC. The EFL_T_ patients also exhibited a significant increase in PaCO_2_ and a decrease in PaO_2_ during peak exercise. O'Donnell et al. [16] extended the findings of Díaz et al. [14, 15] reporting that since the pathophysiological hallmark of COPD is EFL (occurring during exercise and in the advanced disease even at rest), the latter promoted DH which was correlated best with resting IC. DH curtailed V_T_ response to exercise. The inability to expand V_T_ in response to increasing ventilatory demand contributed to exercise intolerance in COPD.

The main finding of these studies was that detection of tidal EFL plays an important role in identifying the factors that limit exercise tolerance because resting EFL clearly separates two populations of patients with significant differences in exercise tolerance. More importantly, their detection provides useful information about the mechanisms limiting exercise tolerance. The detection of EFL during exercise should be carried out also using the NEP technique, as the conventional method for detecting flow limitation based on comparison of tidal with maximal flow-volume curves is not reliable [17]. In the presence of tidal EFL, DH appears to be the main determinant of exercise performance and the magnitude of resting IC, a well-recognized marker of DH, the best clinical predictor [14, 17]. 

EFL may be absent at rest but can be developed and hence detected during any exercise level by the use of NEP. That explains the fact that COPD patients, who are not hyperinflated at rest, develop DH during exercise [17]. It should be noted here that there are instances when DH (reflected by a reduced IC) can occur in the absence of tidal EFL [18, 19], and the presence of tidal EFL may not necessarily result in DH if the available expiratory flow is sufficient to sustain resting ventilation without the need to increase EELV. This is reflected by the fact that there are patients with EFL_T_ and normal IC. Thus, measurement of IC and detection of EFL are complimentary ways for assessing bronchodilator and exercise responsiveness in COPD patients.

 It was found that almost all COPD patients who require mechanical ventilation are flow-limited over the entire range of tidal expiration and that the supine posture promotes flow limitation [20]. 

Despite these potentially adverse consequences of EFL, its prevalence has not been extensively studied until recently, probably due to the lack of simple and noninvasive techniques. The aim of this work was to review the existing physiological techniques of assessing tidal expiratory flow limitation (EFL_T_).

## 3. Oesophageal Balloon Techniques

### 3.1. Fry Method

The definition of EFL implies that a further increase in transpulmonary pressure will cause no further increase in expiratory flow [21]. Therefore, direct assessment of expiratory flow limitation requires determination of iso-volume relationships between flow and transpulmonary pressure (*F*-*P*). Fry et al. [22] were the first who developed such curves in 1950s. The explanation of an isovolumic pressure flow curve lies in understanding its construction. Flow, volume and oesophageal pressure (Poes) are measured simultaneously during the performance of repeated expiratory vital capacity efforts by a subject seated in a volume body plethysmograph, in which gas compression artifact is corrected. The subject is instructed to exhale with varying amounts of effort that are reflected by changes in Poes. From a series of such efforts (~30), it is possible to plot flow against Poes at any given lung volume [21]. The flow reached a plateau at a low positive pleural pressure and that once maximum flow for that volume is reached, it remains constant despite increasing Poes by making expiratory efforts of increasing intensity.

### 3.2. Mead-Whittenberger's Method

The Mead-Whittenberger method [23] directly relates alveolar pressure to flow. Mead-Whittenberger's graphs can be obtained by plotting the flow measured at the airway opening versus the resistive pressure drop during a single breath. In such a way the phenomenon of flow limitation is documented. 

These techniques are technically complex and time consuming. Furthermore, these techniques are invasive because they require the insertion of an oesophageal balloon [22, 23].

### 3.3. Conventional (Hyatt's) Method

Until recently, the “conventional” method used to detect expiratory flow-limitation during tidal breathing was the one proposed by Hyatt [24] in 1961. It consists in superimposing a flow volume loop of a tidal breath within a maximum flow-volume curve. This analysis and the “concept of EFL” have been the basics for understanding respiratory dynamics. Flow limitation is not present when the patient breaths tidally below the maximal expiratory flow-volume (MEFV) curve. According to this technique, normal subjects do not reach flow limitation even at maximum exercise [1, 25]. In contrast, flow limitation is present when a patient breathes tidally along or higher than the MEFV curve. Patients with severe chronic obstructive pulmonary disease (COPD) may exhibit flow limitation even at rest, as reflected by the fact that they breathe tidally along or above their maximal flow-volume curve [1, 21–25]. However, the conventional method to detect flow limitation based on comparison of maximal to tidal expiratory flow-volume curves suffers from several methodological deficiencies. These include the following.

(a) *Thoracic Gas Compression Artefacts.* Volume should be measured with a body-box, instead of using, as is common practice, a pneumotachograph or a spirometer in order to minimize such errors [26]. Consequently, in practice, flow limitation can be assessed only in seated subjects at rest.

(b) *Incorrect Alignment of Tidal and Maximal Expiratory F-V Curves.* Such alignment is usually made considering the total lung capacity (TLC) as a fixed reference point. This assumption may not always be valid [27, 28].

(c) *Effect of Previous Volume and Time History.* Comparison of tidal and maximal *F-V* curves is incorrect, since the previous volume and time history of a spontaneous tidal breath is necessarily different from that of an FVC manoeuvre. Therefore, it is axiomatic that comparison of tidal with maximal *F-V* curves is problematic. In fact, there is not a single maximal *F-V* curve but rather a family of different curves, which depend on the time course of the inspiration preceding the FVC manoeuvre [29–31].

(d) *Respiratory Mechanics and Time Constant Inequalities.* These are different during the tidal and maximal expiratory efforts again making comparisons of the two *F-V* curves problematic [32–34].

(e) *Exercise.* Exercise may result in bronchodilation or bronchoconstriction and other changes of lung mechanics, which may also affect correct comparisons of the two *F-V* curves [35].

(f) *Patient's Cooperation.* Another important limitation of the conventional method is that it requires patient's cooperation. This is not always feasible [27, 28]. 

In fact, it has been clearly demonstrated in several studies [11, 17, 36, 37] comparing the NEP with the conventional technique that the latter is not accurate. As a result, the use of the conventional method is no longer recommended. 

## 4. The Negative Expiratory Pressure (NEP) Technique

In order to overcome these technical and conceptual difficulties, the *negative expiratory pressure (NEP) technique* has been introduced [10, 17, 27, 36]. The NEP technique has been first applied and validated in mechanically ventilated ICU patients by concomitant determination of isovolume flow-pressure relationships [38]. This method does not require performance of FVC manoeuvres, collaboration on the part of the patient, or use of a body-box. It can be used during spontaneous breathing in any body position [39], during exercise [17, 40], and ICU settings [20]. With this technique the volume and time history of the control and test expiration are axiomatically the same.

**Figure 1:**
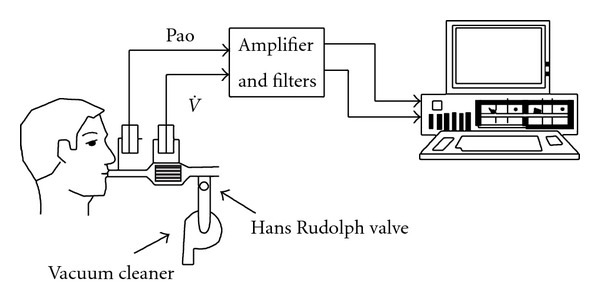
Schematic diagram of equipment setup. Pao: pressure at the airway opening; $\dot{V}$: gas flow (from [36]).

Briefly, a flanged plastic mouthpiece is connected in series to a pneumotachograph and a T-tube (Figure 1). One side of the T-tube is open to the atmosphere, whilst the other side is equipped with a one-way pneumatic valve, which allows for the subject to be rapidly switched to negative pressure generated by a vacuum cleaner or a Venturi device. The pneumatic valve consists of an inflatable balloon connected to a gas cylinder filled with pure helium and a manual pneumatic controller. The latter permits remote-control balloon deflation, which is accomplished quickly (30–60 ms) and quietly, allowing rapid exposure to negative pressure during expiration (NEP). Alternatively, a solenoid rapid valve can be used. The NEP (usually set at about −5 cm H_2_O) can be adjusted with a potentiometer on the vacuum cleaner or by controlling the Venturi device. Airflow ($\dot{V}$) is measured with the heated pneumotachograph and pressure at the airway opening (Pao) is simultaneously measured through a side port on the mouthpiece (Figure 1). Volume (*V*) is obtained by digital integration of the flow signal, and correction of electrical drift is mandatory [36]. While performing the testing, the subjects should be watched closely for leaks at the mouthpiece. Only those tests, in which there is no leak, are valid [41]. Tidal EFL is assessed while seated upright in a comfortable chair or if needed lying supine on a comfortable couch, at least 2 h after eating or taking coffee. Patients are asked to breathe room air through the equipment assembly with the noseclip on (Figure 1). Each subject has an initial 10–15 min trial run, in order to become accustomed to the apparatus and procedure. The flow, volume, and pressure are continuously monitored on the computer screen. When regular breathing is resumed, a series of test breaths are performed with regular breaths in between the test breaths, in which NEP is applied at the beginning of expiration and maintained throughout the ensuing expiration [36]. 

The NEP technique is based on the principle that in the absence of flow limitation, the increase in pressure gradient between the alveoli and the airway opening caused by NEP should result in increased expiratory flow. By contrast, in flow-limited subjects application of NEP should not change the expiratory flow. Our analysis essentially consists in comparing the expiratory $\dot{V}$-*V* curve obtained during a control breath with that obtained during the subsequent expiration in which NEP is applied [36]. 

**Figure 2:**
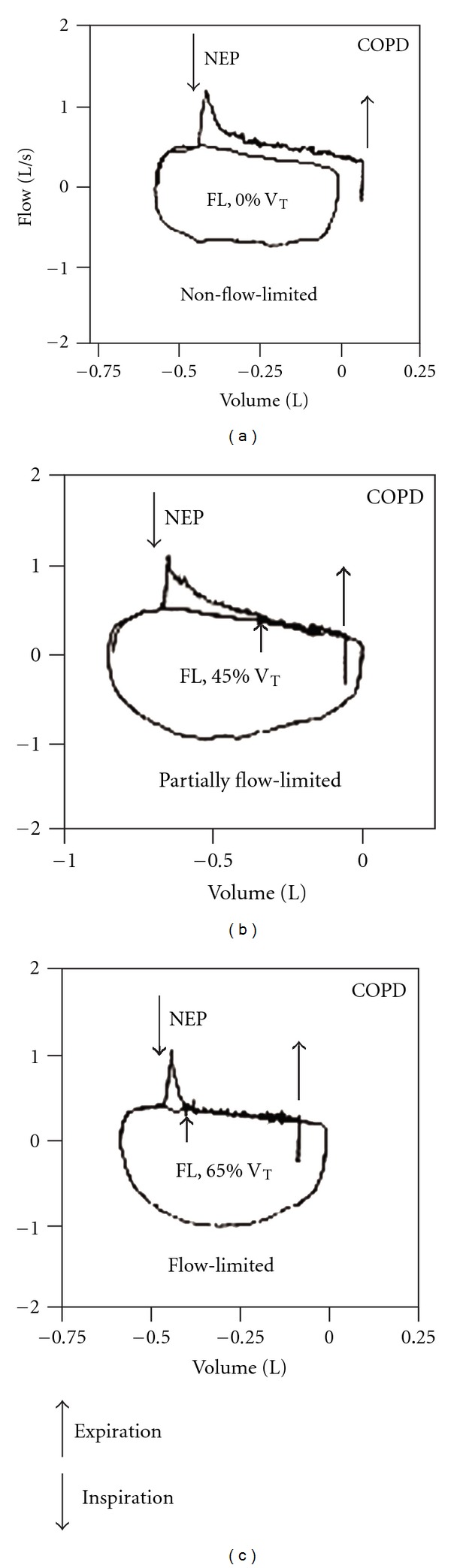
Flow-volume loops of test breaths and preceding control breaths of three representative COPD patients with different degrees of flow-limitation: not flow-limited (NFL) (a), flow-limited (EFL) over less than 50% V_T_ (b), and flow-limited from peak expiratory flow (EFL) (c). Arrows indicate points at which NEP was applied and removed (modified from [10]).

Subjects in whom application of NEP does not elicit an increase of flow during part or all of the tidal expiration (Figures 2(b) and 2(c)) are considered flow-limited (EFL). By contrast, subjects in whom flow increases with NEP throughout the control tidal volume range (Figure 2(a)) are considered as non-flow-limited (non-EFL_T_). If tidal EFL is present when NEP is applied, there is a transient increase of flow (spike), which mainly reflects sudden reduction in volume of the compliant oral and neck structures. To a lesser extent a small artefact due to common-mode rejection ratio of the system of measuring flow may also contribute to the flow transients [10, 36]. Such spikes are useful markers of EFL.

The degree of flow limitation can be assessed using three different EFL_T_ indices: (a) as a continuous variable expressed as % V_T_ in both seated and supine positions (Figure 2) [36], (b) as a discrete variable in the form of three categories classification, that is, non-EFL_T_ both seated and supine, EFL_T_ supine but not seated, and EFL_T_ both seated and supine [36], and (c) as discrete variable in the form of the five-category classification (5-point EFL_T_ score) [10]. 

 In all studies employing the NEP technique, the latter was not associated with any unpleasant sensation, cough, or other side effects [10, 17, 36]. The finding of O'Donnell et al. [42] that application of −9.7 cm H_2_O/L/s of expiratory assistance for 4 min during inspiration and expiration caused unpleasant respiratory sensation can be attributed to negative pressure application differences, that is, NEP, usually at −5 cm H_2_O level, is applied only during expiration every 5–10 breaths intervals.

The use of the NEP technique during tidal flow-volume analysis studies has led to realization of the important role of expiratory flow limitation in exertional dyspnoea and ventilatory impairment for a surprisingly wide range of clinical circumstances, for example, before and after bronchodilation, exercise, ICU, and heliox administration at rest and during exercise [8, 43, 44]. Up to date, no study has questioned reliability and accuracy of the NEP technique. Currently, therefore, the NEP technique can be regarded as the new gold standard to detect EFL_T_, if one takes into account the pros and cons of each available technique. It is a novel, simple, non-invasive, useful research and clinical lung function tool.

## 5. Submaximal Expiratory Manoeuvres

Pellegrino and Brusasco [45] proposed an alternative technique to detect expiratory flow limitation. EFL_T_ was inferred from the impingement of the tidal flow-volume loop on the flow recorded during submaximally forced expiratory manoeuvres initiated from end-tidal inspiration in a body-box. After regular breathing with no volume drift, the subject performs a forced expiration from end-tidal inspiration without breath holding (partial expiratory manoeuvre). Care is taken to coach the subjects not to slow down the inspiration preceding the partial forced manoeuvre, thus minimizing the dependence of forced flows on the time of the preceding inspiration. A deep inspiration to TLC recorded soon after the gentle forced manoeuvre allowed the loops to be superimposed and compared at absolute lung volume. Flow limitation is defined as the condition of tidal expiratory flow impinging on the maximal flow generated during the gentle forced expiratory manoeuvre. Since this method requires a body box measurements cannot be made in different body postures, ICU, or during exercise testing.

## 6. Squeezing the Abdomen during Expiration

Workers in Brussels have shown that manual compression of the abdomen coinciding with the onset of expiration can be used as a simple way of detecting flow limitation at rest [46] and during exercise [47]. With one hand placed on the lower back of the patient and other applied with the palm at the level of the umbilicus perpendicular to the axis between the xiphoid process and the pubis, the operator first detects a respiratory rhythm by gentle palpation, and then after warning the subject applies a forceful pressure at the onset of expiration. As in the NEP technique, the resulting expiratory flow-volume loop recorded at the mouth is superimposed on the preceding tidal breath. Failure to increase expiratory flow indicates flow limitation. This technique produces clear differences between normal subjects and patients with COPD. The presence of flow limitation during exercise detected during exercise in COPD patients was associated with increases in the end-expiratory lung volume (EELV) [47]. Interestingly, not all subjects with COPD exhibited flow limitation when lung volume changed, a finding which requires confirmation. The method is appealingly simple, not influenced by the upper airway compliance, and like the NEP method, it avoids problems with the preceding volume history of the test breath. Despite initial concerns about the possibility that gas compression in the alveoli would produce false positive results, this does not seem to be a practical problem. However, unlike the NEP method, it is virtually impossible to squeeze at the precise of expiration. Thus far this technique has not been widely applied despite its relative simplicity.

## 7. Forced Oscillation Technique (FOT)

Another approach for detecting EFL_T_ has been the forced oscillation technique (FOT) previously applied to look at the frequency dependence of resistance in a range of lung diseases and now available commercially in a modified form using impulse oscillometry [48, 49]. The principle here is that flow limitation will only be present in patients with obstructive pulmonary disease during expiration. Normally oscillatory pressures generated by a loud-speaker system at the mouth are transmitted throughout the respiratory system, and by studying the resulting pressures which are in and out of phase with the signal, both the respiratory system resistance and reactance (a measure of the elastic properties of the system) can be computed. When flow limitation occurs, wave speed theory predicts that a choke point will develop within the airway subtended by that “unit” of the lung. In these circumstances, the oscillatory pressure applied at the mouth will no longer reach the alveoli and the reactance will reflect the mechanical properties of the airway wall rather than those of the whole respiratory system. As a result, reactance becomes much more negative and there is a clear within breath difference between inspiration and expiration. Dellacá and colleagues [49] used this property to investigate the distribution of changes in intrabreath reactance in normal subjects and COPD patients who were instrumented with balloon catheters. In a recent study Dellacá et al. [50] found a good agreement between NEP and FOT despite the fact that the FOT method may detect regional as well as overall EFL_T_. NEP detects the condition in which all possible pathways between airway opening and the alveoli are choked. When this occurs, the total expiratory flow is independent of the expiratory pressure, a condition of “global” expiratory flow limitation. In contrast, FOT assesses the amount of the lung that is choked during expiration only. This measures “regional” flow limitation, and a threshold value may indicate when the regional flow limitation reaches the condition of “global” flow limitation. Therefore, when “global” expiratory flow limitation is reached, the two techniques should produce the same response [50].

It does appear to hold considerable promise, but to date, only a few studies to detect EFL_T_ with this method have been reported. On the other hand, FOT is very complex, expensive as it requires the special FOT equipment, and time consuming.

## 8. Technegas Method

 Technegas is an aerosol of ^99m^Tc-labeled carbon molecules with small diameter (<0.01 *μ*m) [19] capable of depositing even in the most peripheral regions of the lung. Pellegrino et al. [19] used the inhalation of Technegas to reveal sites (“hot spots”) of EFL_T_ after induced bronchocontsriction in asthmatic patients. During forced expiration, the flow-limiting segment is known to be located first in the large intrathoracic airways and then to move peripherally. However, the present scintigraphic technique cannot precisely define the anatomical location of the flow-limiting segment during tidal breathing. Therefore, what the “hot spots” represent appears to be uncertain. The authors claim that this technique is useful to detect “regional” EFL_T_ well before the NEP and submaximal expiratory manoeuvre techniques.

## 9. Breath-by-Breath Method

 The most recent method to detect EFL_T_ is the one using breath-by-breath quantification of progressive airflow limitation during exercise applied in stable COPD patients [51]. The authors have noted that during heavy exercise in COPD patients, dynamic airways compression leads to a progressive fall in intrabreath flow. This is manifested by an increasing concavity in the spontaneous expiratory flow-volume (SEFV) curve. The new method consists in quantifying the SEFV curve configuration breath-by-breath during incremental exercise utilizing a computerized analysis. For each breath's SEFV curve, points of highest flow and end-expiration were identified to define a rectangle's diagonal. Fractional area within the rectangle below the SEFV curve was defined as the “rectangular area ratio” (RAR). RAR < 0.5 signifies concavity of the SEFV curve. However, this method may be useful only during exercise because inspection of SEFV curve during resting breathing is not a reliable means in detecting EFL_T_ [41]. Severe COPD patients often exhibit a mechanically active expiration, which is characterized by abdominal activity. This necessarily affects the shape of SEFV curve, making it concave with respect to the volume axis, even in the absence of EFL_T_ [52].

## 10. Conclusions

 The newer aforementioned techniques represent a substantial advance on traditional approaches which compared tidal and maximal flow-volume loops or even the more robust but time-consuming method of determining partial expiratory flow-volume loops. By freeing both parts, the doctor and the patient, from the limitations of the oesophageal balloon catheters and body plethysmograph, they have opened up a new era in understanding modern physiological principles like the tidal expiratory flow limitation [8, 43, 44]. Among the available physiological techniques to detect EFL_T_, the NEP should probably be regarded as the new gold standard. This view is supported by the data obtained from the NEP's application in a wide variety of settings [8, 43, 44]. However, extensive comparisons between these different methods are needed before the best “test” or combination of techniques can be unequivocally recommended to correctly assess EFL_T_.
